# Fracture Resistance of Resin Matrix Ceramic Post and Core Compared to Prefabricated Fiber-Reinforced Composite Post and Core in Non-Ferrule Effect Teeth: An *In* *Vitro* Study

**DOI:** 10.1055/s-0044-1789001

**Published:** 2025-03-13

**Authors:** Keeratikarn Kunawongkrit, Basel Mahardawi, Pheeradej Na Nan, Palawat Laoharungpisit, Kwanchanok Ratanakupt, Napapa Aimjirakul

**Affiliations:** 1 Department of Conservative Dentistry and Prosthodontics, Faculty of Dentistry, Srinakharinwirot University, Bangkok, Thailand; 2 Department of Oral and Maxillofacial Surgery, Faculty of Dentistry, Chulalongkorn University, Bangkok, Thailand; 3 National Cyber Security Agency, Bangkok, Thailand; 4 Department of General Dentistry, Faculty of Dentistry, Srinakharinwirot University, Bangkok, Thailand

**Keywords:** ceramic, *invitro* technique, post and core, resin matrix

## Abstract

**Objectives:**

This research studies the fracture resistance of a non-ferrule endodontically treated tooth restored with two types of resin matrix ceramic (Enamic and Cerasmart) post and core compared with the conventional prefabricated fiber-reinforced composite (FRC) post and core.

**Materials and Methods:**

Thirty single-root-canal premolars were sectioned to 13 mm root length and then all the roots were filled, using a crown-down technique for root canal preparation and one cone technique for root canal obturation, All the roots were randomly divided into three groups ( *n* = 10) according to post and core material; (1) Enamic group (EN), (2) Cerasmart group (CM), and (3) prefabricated fiber post group (FRC). Three groups of specimens were tested using a universal testing machine (EZ Test Series, Shimadzu, Japan). Specimen blocks were fixed into a holder with an inclination of 45 degrees. The force was loaded to a palatal incline of buccal cusp at a crosshead speed of 0.5 mm/minute until there was split or fracture of the tooth. The maximum braking force was recorded in Newton (N), and the mode of failure was observed by a stereomicroscope.

**Statistical Analysis:**

The dependent variable was fracture resistance (maximum breaking force) and the data were analyzed using one-way analysis of variance and then multiple comparison Tukey's tests were used, aiming to find means that are significantly different among the groups. Moreover, the failure mode was analyzed using the chi-square test.

**Results:**

A significant difference was recorded. Teeth restored with prefabricated FRC had the most fracture resistance 342.19 ± 79.34 N ( *p* = 0.007), followed by the resin matrix ceramic group (265.10 ± 48.58 N: Cerasmart) and Enamic group (260.98 ± 43.96 N: Enamic). No significant difference between the Enamic and Cerasmart groups was noted. Additionally, no significant difference in the mode of failure was recorded among the three groups.

**Conclusion:**

The fracture resistance of a non-ferrule endodontically treated tooth restored with the conventional prefabricated FRC post and core is greater than that restored with either type of resin matrix ceramic (Enamic and Cerasmart). This explains the higher failure rate resulting from the use of resin matrix ceramics.

## Introduction

Endodontically treated teeth should be restored due to the weakness of the tooth that occurs from many factors such as alteration of tooth structure, properties, and sensation. The options for restoration are a crown, or post and core with a crown, depending on certain aspects such as the remaining tooth structure, as well as the position of the tooth and its function. 1 2 3 It was found that most of the failure of endodontically treated teeth is for prosthodontic reasons. 4

The post or dowel is the restorative choice for endodontically treated teeth, the main role of the post is to retain the core to the root. In the past, casting alloy post and core was the most popular, but the limitation of casting alloy was the lack of flexibility compared with the dentine, which can lead to a root fracture, in addition to the overall complex procedure. 5 6 As a result, casting alloy post and core was later replaced by prefabricated fiber-reinforced composite (FRC) post and core that has more flexibility and is easy to use. However, FRC post has less strength, especially in a non-ferrule tooth, 7 8 The effect of the post on non-ferrule teeth is still unclear but the result of some studies showed that the post led to catastrophic failure, emphasizing that fiber post cannot replace the lack of ferrule. 9 10 The “ferrule” is the coronal tooth structure that is 1.5 to 2 mm in height and thickness. The lack of ferrule will lead to biomechanical failure. The reason behind this incident is mainly the insufficient bond strength of the abutment to resist fracture of the tooth, as well as the post and core. 11 The fracture resistance of prefabricated FRC post is still inadequately explored, especially in compromised ferrule teeth. A previous study performed in upper anterior endodontically treated teeth found that prefabricated quartz fiber post has a good fracture resistance in 360-degree ferrule teeth and teeth that have a ferrule at the palatal side (occlusal loaded side), whereas teeth that have a ferrule only labial side greatly reduced the fracture resistance, and the lowest incidence was found in a tooth with no ferrule. 7

Post and core that are fabricated with computer-aided design/computer-aided manufacturing (CAD/CAM) technique is a new interesting choice because there is more strength and more attachment to the root canal wall. 12 13 Resin matrix ceramic is the one that can be fabricated with CAD/CAM technique and has good properties for post and core, since it possesses both advantages of ceramic and composite material; flexural strength is approximate to dentine (16–20 GPa), and have a tooth-like color. 14 15 16 17 18 Vita Enamic and Cerasmart are resin matrix ceramics but not in the same subgroups. Vita Enamic is a glass ceramic in a resin interpenetrating matrix or polymer infiltrated ceramic network (PICN) subgroup, while Cerasmart is a resin nanoceramic subgroup. This subgroup of material contains a highly cured resin matrix. The matrix is reinforced with nanoceramic particles that take approximately 80% of its weight. The particles are a combination of various nanoceramic particles such as discrete silica nanoparticles, zirconia nanoparticles, and zirconia-silica nanoclusters. 19

To date, there is still no study that assesses the strength of the previously mentioned types and compares them to the conventional post and core. Therefore, this study aimed to compare the fracture resistance of a non-ferrule endodontically treated tooth restored with two subgroups of resin matrix ceramic (Enamic and Cerasmart) post and core compared with the conventional prefabricated FRC post and core. It was thought that the post and core made from resin matrix ceramic could potentially demonstrate acceptable performance and overcome the disadvantages of the casting alloy and FRC post. The null hypothesis was that there is no statistically significant difference in the fracture resistance of non-ferrule endodontically tooth restored with Vita Enamic or Cerasmart, and prefabricated FRC.

## Materials and Methods

### Sample Size Calculation

The sample size was calculated by using the G Power 3.1.9.6 program. The effect size was calculated from the study of Falcão Spina et al in 2017, 15 obtaining a value of 0.91. The power was set at 0.95, with an *α* level of 0.05. This resulted in a final sample size of 24. Considering possible technical issues in sample preparation, this was increased to 30 ( *n* = 10 for each group).

### Sample Selection and Collection

Thirty premolars were extracted and stored in thymol 0.1% solution. The sample teeth did not have large dental caries, restoration material, or severe damage to coronal and root structure. All teeth had one straight root canal and were as similar in size as possible. The teeth were observed under a stereomicroscope, and periapical radiographs were taken. All teeth were divided into three groups randomly (FRC = prefabricated fiber post group, EN = Vita Enamic group, CM = Cerasmart group). This study was approved by the Human Research Ethical Committee of Srinakharinwirot University (SWUEC-661006).

### Sample Preparation

#### Tooth Preparation

All roots were lined with pink wax with a thickness of 0.5 to 1 mm and then were fixed into blocks with self-polymerized acrylic resin (Unifasttrad; GCchemicals, Japan). The margin emerged above the block approximately 3 mm to perform a biologic width. Roots were stored in room temperature water during the curing period, for protection from heat damage. After polymerization, pink wax was removed from the roots by using hot water and was replaced with a light body polyvinyl silicone impression to mimic periodontal ligament. Then, the roots were returned to their blocks.

**Fig. 1:**
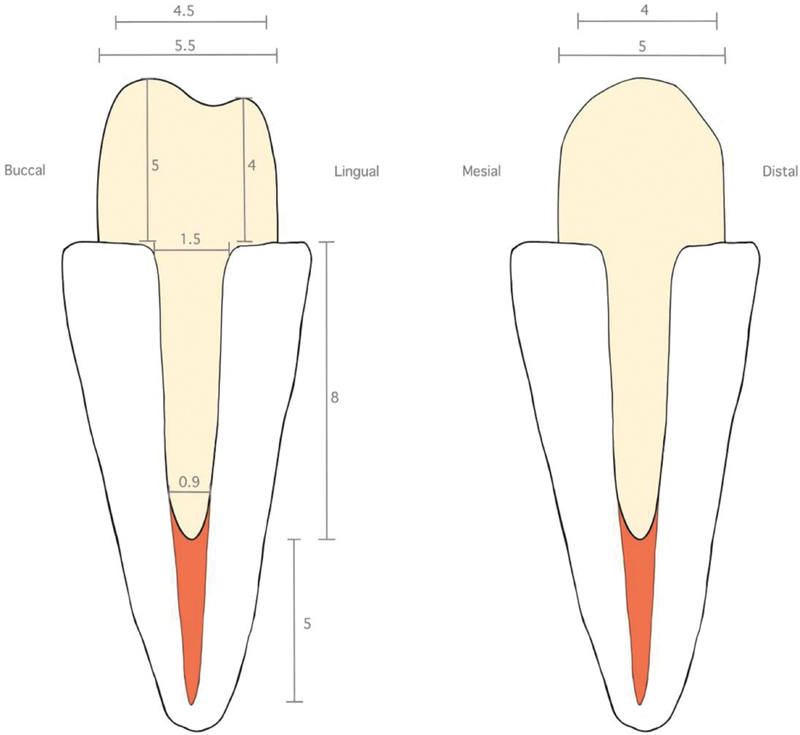
Post and core preparation.

Crowns were sectioned horizontally for a standardized 13 mm root length by a saw machine. 15 Root canal treatment was done for all teeth using a rotary file (ProTaper Next files) form no. X1-X3 with a crown-down technique and irrigation with 2.5% sodium hypochlorite (NaOCl) solution and 17% solution ethylenediaminetetraacetic acid. The root canals were dried and obturated with gutta-percha and a sealer using a matching cone (ProTaper Next Conform Fit Gutta Percha Points X3) and warm vertical compaction technique. Specimens were stored in thymol 0.1% solution for 1 week before the canal was drilled. After 1 week, all roots were drilled by a Peeso reamer. Gutta-percha was removed 8 mm to leave an apical seal for 5 mm. The length of the post space was 8 mm, and the diameter was 0.9 mm apically and 1.5 mm coronally (the shape of the post space was controlled by the size of the post drill), shown in Fig. 1 . A canal impression of the EN and CM groups was done with polyvinyl siloxane (3M ESPE Express XT VPS Light Body Impression) and the impression was sent to the dental laboratory for scanning. In the FRC group, all roots were scanned directly with a laboratory scanner (3Shape E4).

#### Post and Core Fabrication

**Fig. 2:**
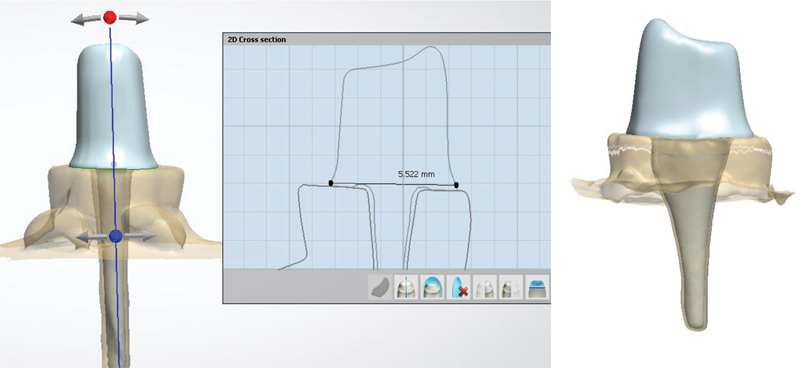
Post and core were designed in the program TRIOS Design Studio.

**Fig. 3:**
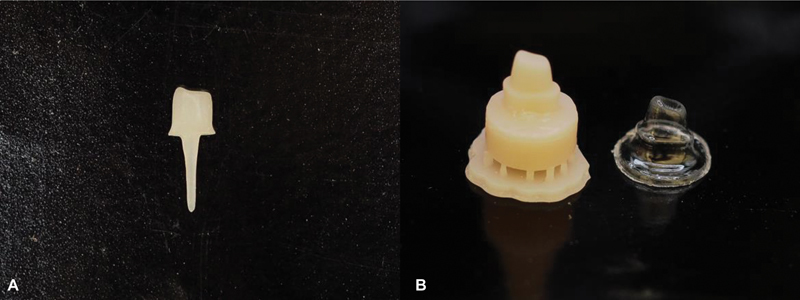
( **A** ) Post and core fabricated by milling technique. ( **B** ) Resin die and plastic mold.

Post and core were designed with a software program (TRIOS Design Studio, 3Shape TRIOS). The core was fabricated utilizing a similar manner, and the measurements were standardized: buccal height (5 mm), palatal height (4 mm), bucco-palatal width cervically (5.5 mm) and incisally (1 mm) each cusp, and mesiodistal width cervically (5 mm) and incisally (4.5 mm) ( Fig. 2 ). Then, the Enamic and Cerasmart post and core were fabricated by a milling technique. The resin dies of the FRC group were printed for the fabrication of a plastic mold by a suck-down vacuum technique ( Fig. 3 ).

#### Post and Core Cementation

Cementation of EN and CM groups was done with self-etching resin cement (Panavia V5). Before cementation, the post surface was etched with 5% hydrofluoric acid for 60 seconds, rinsed, and dried, and then a ceramic primer, containing silane coupling agents (Clearfil ceramic primer plus), was applied. The tooth primer (Panavia V5 tooth primer) was applied to the root canal surface. Resin cement was loaded to the canal by a canal-size mixing tip, the post was gently seated to the matching canal with forceps and finger pressure, and then light-cured with an light-emitting diode (LED) curing light.

In the FRC group, prefabricated FRC posts (DT light posts) were fixed with core built-up material (Clearfil DC core plus) and built up the core by using a clear plastic mold. Before cementation universal bonding agent (Clearfil S ^3^ bond Universal quick) was applied to the root canal surface, then dry and light cured. Core built-up material was loaded to the canal by a canal-size mixing tip, the post was seated to the matching canal gently by forceps and finger pressure and light curing with an LED curing light, the core built-up material was loaded to a clear plastic mold, then a plastic mold was seated to the matching root and light curing with an LED curing light. All procedures followed the manufacturer's suggestion.

#### Crown Fabrication and Cementation

All specimens were scanned with a laboratory scanner and digitally designed to fabricate full metal crowns. The anatomy of the crowns was standardized by using CAD design and wax printing and casting techniques. The crowns were fabricated into lower premolar form, then were cemented with their matching roots using self-etching resin cement (Panavia V5). All procedures followed the manufacturer's suggestion.

### Testing and Data Collecting

**Fig. 4:**
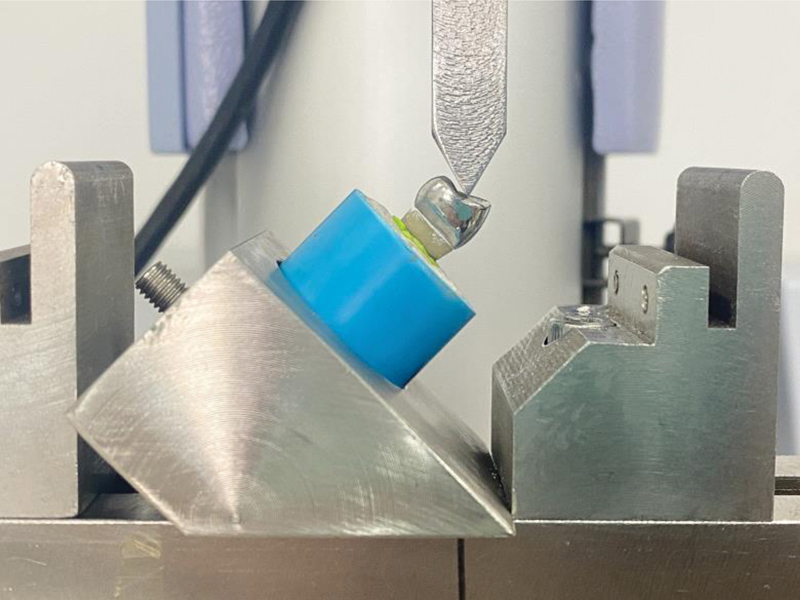
Specimens were tested using a universal testing machine (EZ Test Series, Shimadzu, Japan).

Three groups of specimens were tested using a universal testing machine (EZ Test Series, Shimadzu, Japan). Specimen blocks were fixed into a holder, with an inclination of 45 degrees (angle of 135 degrees to the long axis of the crown on the palatal incline of buccal cusp), 14 shown in Fig. 4 . The force was loaded to a palatal inclination of buccal cusp at a crosshead speed of 0.5 mm/minute until there was split or fracture of the root. 15 The maximum braking force was recorded in a Newton (N), and the mode of failure was observed by stereomicroscope and interpreted, as explained in Table 1 .

**Table 1:** Classification of the mode of failure

Failure type	Description
Type 0	Absence of visual damage
Type 1	Fracture, crack, or chip up to 50% of the coronal portion of the core or crown, without involvement of the post portion
Type 2	Fracture, crack, or chip of more than 50% of the coronal portion of the core or crown, without involvement of the post portion
Type 3	Fracture or crack of the coronal portion of the core, with the involvement of the post portion, but without involvement of the root dentin
Type 4	Any type of fracture involving the root dentin at the cervical third
Type 5	Any type of fracture involving the root dentin at the middle or apical thirds

## Statistical Analysis

The statistical analysis was conducted using the Statistical software SPSS Statistics 29.0.1.0 (SPSS Inc., Illinois, United States). The test of normality was performed using the Shapiro–Wilk test due to the small number of specimens, *n* < 50. The dependent variable was fracture resistance (maximum breaking force) and the data were analyzed using one-way analysis of variance (ANOVA) and then multiple comparison Tukey's tests were used, aiming to find means that are significantly different among the groups. Moreover, the failure mode was analyzed using the chi-square test. The tests were performed at a confidence level of 95% and with a *p* -value of < 0.05 to represent statistical significance.

## Results

The results of the Shapiro–Wilk test of means fracture resistance of endodontically treated tooth showed that all groups had a normal distribution ( *p* -value > 0.05), and the result of the homogeneity test with Levene statistic indicated that all groups of data had no difference between the populations ( *p* -value > 0.05). Therefore, one-way ANOVA could be used.

Means of fracture resistance (maximum breaking force) of a non-ferrule endodontically treated tooth restored with two types of resin matrix ceramic (Enamic and Cerasmart) post and core or restored with conventional prefabricated FRC post and core are shown in Table 2 . The ANOVA results show significant differences between the three groups ( *p* = 0.007). Teeth restored with prefabricated FRC had the highest fracture resistance, followed by the resin matrix ceramic group (Enamic and Cerasmart). No significant difference between Enamic and Cerasmart groups was recorded.

**Table 2:** Mean of fracture resistance

Type of restorations	*n*	Mean	SD	Min	Max
Enamic (EN) a	10	260.975	43.959	173.1	331.8
Cerasmart (CM) a	10	265.102	48.577	174.6	331.02
Prefabricated fiber-reinforced composite (FRC) a	10	342.195	79.339	224.27	454.6

Abbreviation: SD, standard devi8ation.

a The same superscript letters show no significant differences.

**Fig. 5:**
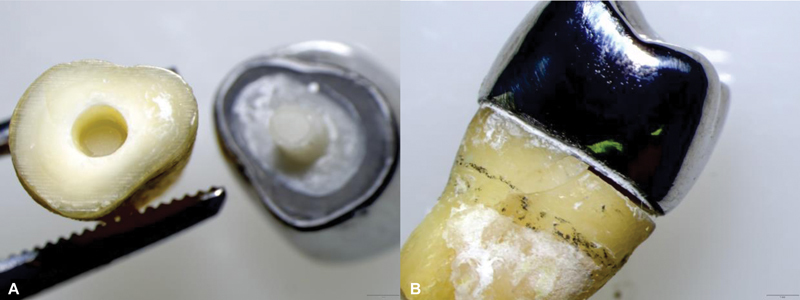
( **A** ) Type 2 failure. ( **B** ) Type 4 failure.

The mode of failure of each group is shown in Table 3 . Most of the teeth failed in type 4 condition: the fracture involving the root dentin at the cervical third. The restorable condition and (type 1, 2, 3) had 10 teeth, distributed among EN, CM, and FRC groups. Type 5 is the most catastrophic failure and was seen in 2 teeth in the CM and FRC groups ( Fig. 5 ).

**Table 3:** The mode of failure of each group

	Restorable conditions				Unrestorable conditions	
	Type 0	Type 1	Type 2	Type 3	Type 4	Type 5
EN	0	0	3	1	6	0
CM	0	2	0	1	6	1
FRC	0	1	2	0	6	1

Abbreviations: CM, Cerasmart; EN, Enamic; FRC, fiber-reinforced composite.

Failure mode distribution is presented in Table 4 . Restorable failure patterns were 40% in the EN group, 30% in the CM group, and 30% in the FRC group, while unrestorable failure patterns were 60, 70, and 70% in the EN group, CM group, and FRC group, respectively. There was no significant difference among the three groups ( *p* -value = 0.861).

**Table 4:** Results of the chi-square test of the failure mode

	EN, *n* (%)	CM, *n* (%)	FRC, *n* (%)	*p* -Value
Restorable	4 (40)	3 (30)	3 (30)	0.861
Unrestorable	6 (60)	7 (70)	7 (70)	

Abbreviations: CM, Cerasmart; EN, Enamic; FRC, fiber-reinforced composite.

## Discussion

The results of our study show that non-ferrule endodontically treated teeth restored with conventional prefabricated FRC post and core have the highest fracture resistance when testing with the universal testing machine. At first, it was assumed that the resin matrix ceramic groups may have a higher or equal fracture resistance to the FRC group, but the results demonstrated that prefabricated FRC post and core have more strength than other groups. Therefore, the null hypothesis was rejected. The reason could be that DT-light post have a flexural strength greater than Enamic and Cerasmart (DT light post: 723.24 MPa, 20 Cerasmart: 216.5 Mpa, Enamic: 148.7 MPa). 16 No significant difference was observed in the fracture resistance between the two resin matrix ceramic groups, possibly because Enamic and Cerasmart have very close mechanical properties, such as flexural strength and fracture toughness. 16 A study of human bite force showed that in an adult individual, this was 205 to 295 N at the molar area, 375 to 475 N in the premolar area, and in the incisal area it ranges between 205 and 295 N. 21 The mean values of maximum fracture resistance of EN, CM, and FRC group are 260, 265, and 342 N, respectively. Based on these results, at the molar and premolar area no group can resist the maximum bite force at the molar area; however, in the incisal area, every group of restoration can resist the maximum bite force. Although the results demonstrated that prefabricated FRC post and core achieved better performance than resin matrix ceramic post and core, in the clinical aspect prefabricated FRC post and core or resin matrix ceramic post and core can be used only in non-ferrule anterior teeth, that is, both may provide overall acceptable clinical performance and durability.

Although most failure incidents of all groups were type 4 (fracture involving the root portion), no fracture of the fiber post in any specimens of the FRC group was noted, and it was limited to the composite core. Based on these results, it is indicated that the fiber post can reinforce and retain the core of the non-ferrule tooth better than the resin matrix ceramic post and core.

The metallic crown, not a ceramic crown, was chosen in this study, attempting to avoid the fracture of the crown before the fracture of the post and core. Another reason was the cost-effectiveness of this material. As for selecting lower premolars, this was due to the fact that it is the most common extracted healthy tooth (for orthodontic reasons), with one root canal, 22 23 therefore matching the conditions of this experiment.

The results of the present study are consistent with the report of Iemsaengchairat and Aksornmuang, 24 who compared the fracture resistance of endodontically treated teeth that have thin walls without ferrules restored, using different materials. The outcomes showed that the teeth restored with multiple DT Light-Post have a greater fracture resistance than the teeth restored with Shofu HC post and core (resin matrix ceramic). Moreover, it was noted that the fiber post has greater resistance than the resin matrix ceramic post and core. However, another previous report 15 25 reached a different conclusion compared with the current study. Falcão Spina et al 15 compared fracture resistance of the teeth restored with Vita Enamic, Lava Ultimate post and core (resin matrix ceramic), and experimental CAD-CAM glass fiber post, and showed that the teeth restored with Lava Ultimate post and core have the highest fracture resistance, with no significant difference between experimental CAD-CAM glass fiber post and Vita Enamic post and core. The reason might be the difference in the type of fiber post. Moreover, Fathey et al 25 compared the fracture resistance of three esthetic CAD-CAM post and core, namely, glass FRC, polyetheretherketone (PEEK), and polymer-infiltrated ceramic network (Vita Enamic). The outcome of this investigation showed that PEEK had less fracture resistance than FRC and Enamic. Additionally, no significant difference between FRC and Enamic was noted. A 2-mm coronal structure of the tooth (ferrule) was left in this previous study, which could be a reason why the result is different from the present experiment. The results of these studies assumed that the FRC post is still a good choice for restoring non-ferrule endodontically treated teeth but in the clinical application when the tooth has no more coronal structure it is hard to build up the hold of abutment with resin composite core, CAD-CAM post and core is the easy way to fabricate the core structure of the tooth with a proper shape and size.

The use of CAD-CAM to fabricate one piece of fiber post and core is interesting too, and several investigations tried to fabricate this kind of post and core. 15 26 Therefore, future studies, with more direct comparisons of different types of posts or composite core such as a bulk-fill composite, 27 would be of high value, and further comparisons of fracture resistance of CAD-CAM one piece fiber post and core to the conventional prefabricated FRC post and core is recommended.

Regarding the clinical application, the choice of CAD-CAM resin matrix ceramic post and core may come from its nature, being easy to design and fabricate to the needed actual shape and size, with a tooth-like color of this material. Although conventional prefabricated FRC post and core has more fracture resistance than CAD-CAM resin matrix ceramic post and core, if upper anterior teeth that were restored with prefabricated FRC post in an anterior deep bite condition, the fiber post could interfere with lower anterior teeth, since the axis of the post must be fixed to the axis of the root canal. Furthermore, aiming to resolve this condition, the clinician may have to prepare the fiber post and reduce its structure, which will lead the post to have less function to retain the core, as well as the difficulty of preserving a proper space for the final crown. In this specific scenario, CAD-CAM resin matrix ceramic post and core can be a good choice, since the clinician has the ability to adjust the angulation of the core, to avoid the interference of lower anterior teeth and keep a proper space for the final crown.

The limitations of the study should be kept in mind when interpreting the results. First, although the size and shape of the teeth were standardized, reaching exact similarity in the size and shape of root canals is not possible. Furthermore, some technical limitations could be thought of, that is, during the digital design of Vita Enamic and Cerasmart post and core, the software program had to compensate for the root canal undercut, thus, the gap between post and root canal is increased.

## Conclusion

Within the limitations of the present experiment, it is concluded that the fracture resistance of a non-ferrule endodontically treated tooth restored with the conventional prefabricated FRC post and core is greater than that restored with two subgroups of resin matrix ceramic (Enamic and Cerasmart) post and core, and most of the failure in every group is an unrestorable condition.
